# Interaction between BEND5 and RBPJ suppresses breast cancer growth and metastasis via inhibiting Notch signaling

**DOI:** 10.7150/ijbs.70866

**Published:** 2022-06-27

**Authors:** Yanzhu Shi, Deyu Zhang, Jingyi Chen, Qiwei Jiang, Songze Song, Yue Mi, Tao Wang, Qinong Ye

**Affiliations:** 1 Medical College, Guizhou University, Guiyang 550025, P.R. China.; 2 Department of Medical Molecular Biology, Beijing Institute of Biotechnology, Beijing 100850, P.R. China.; 3 Jinzhou Medical University, Jinzhou, Liaoning 121001, P.R. China.; 4 Department of Oncology, The fifth Medical Center of Chinese PLA General Hospital, Beijing 100071, P.R. China.

**Keywords:** Breast cancer, metastasis, bioinformatics, BEND5, Notch signaling

## Abstract

High frequent metastasis is the major cause of breast cancer (BC) mortality among women. However, the molecular mechanisms underlying BC metastasis remain largely unknown. Here, we identified six hub BC metastasis driver genes (BEND5, HSD11B1, NEDD9, SAA2, SH2D2A and TNFSF4) through bioinformatics analysis, among which BEND5 is the most significant gene. Low BEND5 expression predicted advanced stage and shorter overall survival in BC patients. Functional experiments showed that BEND5 could suppress BC growth and metastasis *in vitro* and *in vivo*. Mechanistically, BEND5 inhibits Notch signaling via directly interacting with transcription factor RBPJ/CSL. BEN domain of BEND5 interacts with the N-terminal domain (NTD) domain of RBPJ, thus preventing mastermind like transcriptional coactivator (MAML) from forming a transcription activation complex with RBPJ. Our study provides a novel insight into regulatory mechanisms underlying Notch signaling and suggests that BEND5 may become a promising target for BC therapy.

## Introduction

Breast cancer (BC) is one of the most common cancers with the high death rate among women in the world 1, 2. Despite considerable progress in advanced therapy of BC, distant metastasis leads to poor prognosis in patients 3, 4. Therefore, there is an urgent need to explore novel therapeutic targets and mechanisms to prevent BC metastasis.

The abnormal activation of Notch signaling pathway is recognized as an important element of BC metastasis 5-7, which depends on transcription factor recombination signal binding protein for immunoglobulin kappa J region (RBPJ/RBP-Jκ/CSL) 8. When Notch signaling is activated by Notch ligands such as DLL4 and Jagged1, intracellular domain of Notch (NICD) is released and translocated into cell nucleus. Nuclear NICD interacts with RBPJ and recruits mastermind like transcriptional coactivator (MAML) to bind RBPJ. The NICD-RBPJ-MAML ternary complex has ability to transactivate several oncogenes, such as hes family bHLH transcription factor 1 (HES1) and hes related family bHLH transcription factor with YRPW motif 2 (HEY2) 9. Because of the critical role of Notch signaling in cancer, exploring novel molecular targets associated with Notch signaling activity have crucial significance for BC therapy.

The BEN (BANP, E5R and NAC1) domain is a conserved domain and mediates protein-protein and protein-DNA interactions during gene transcription. The interaction between BEN domain of BEN domain containing 3 (BEND3) and TPR domain in PLK1-interacting checkpoint helicase (PICH) can stimulate the activity of PICH, which is required for maintaining genomic stability 10. BEN domain containing 5 (BEND5) belongs to the BEN domain family, and represses target gene transcription via BEND5-DNA binding 11. In colorectal cancer, BEND5 hypermethylation suppresses BEND5 protein expression and promotes cell growth 12. However, whether BEND5 regulates Notch signaling and BC growth and metastasis remains unknown.

In this study, through combination of bioinformatics analysis and functional experiments, we identified BEND5 as a vital BC suppressor gene associated with tumor growth and metastasis* in vitro* and *in vivo*. To understand the mechanisms underlying the function of BEND5 in breast cancer, Gene Set Enrichment Analysis (GSEA) was performed. Notch signaling pathway was significantly enriched. We further demonstrated that BEN domain of BEND5 interacts with NTD domain of RBPJ. BEND5 inhibited Notch signaling activation via preventing RBPJ/MAML interaction.

## Materials and Methods

### Plasmids, cell lines and reagents

The target gene fragments were amplified by PCR and were inserted into pcDNA3.0 (Invitrogen) to construct eukaryotic expression vectors. The target sequence of BEND5 shRNA was 5'-GCAAATACGTCGTCCTATT-3'. The human breast cancer cell lines MDA-MB-231 and ZR75-1, and human embryonic kidney cell line HEK293T were purchased from the American Type Culture Collection. Anti-BEND5 (20931-1-AP), anti-HEY2 (10597-1-AP), anti-E-cadherin (20874-1-AP), anti-N-cadherin (22018-1-AP), anti-Vimentin (10366-1-AP), and anti-MAML1 (55493-1-AP) were purchased from Proteintech. Anti-HES1 (ab71559) was purchased from Abcam. Anti-BEND5 (NBP2-26211) was from Novus Biologicals. Anti-RBPJ (sc-271128), anti-Notch1 (sc-373891), and anti-β-actin (sc-47778HRP) were purchased from Santa Cruz Biotechnology. Anti-FLAG (A8592), anti-FLAG M2 agarose (A2220), anti-c-MYC gel (E6654), and anti-c-MYC-peroxidase (A5598) were from Sigma-Aldrich. Dulbecco's modified Eagle's medium (DMEM, 12491-015) was purchased from Gibco. VigoFect reagent (T001) was purchased from Vigorous Biotechnology. CCK8 reagent (CK04) was purchased from Dojindo. Paraformaldehyde (BL539A) was purchased from Biosharp.

### Data collection

The data of 1109 BC tissue samples and 113 adjacent tissue samples were obtained from The Cancer Genome Atlas (TCGA-BC, www.cancergenome.nih.gov). Gene expression profiles of MDA-MB-231 and its lung metastatic subpopulations were obtained from GSE138122 dataset 13.

### Identification of metastasis driver genes (MDGs)

The limma R package was used to standardize and screen for the differentially expressed genes (DEGs) based on GSE138122 dataset and TCGA-BC dataset. False Discovery Rate (FDR) < 0.05 and |log2 fold change (FC)| > 1 were used as the cut-off threshold. The intersecting DEGs in GSE138122 dataset and TCGA-BC dataset were identified as MDGs.

### Construction and evaluation of BC-specific prognostic model

Due to the small sample size and incomplete clinical information of other BC types, univariable Cox regression analysis was used to identify the relationship between MDGs and overall survival (OS) of 959 patients with infiltrating ductal carcinoma or infiltrating lobular carcinoma from TCGA-BC dataset. In order to establish prognostic signature for each patient, multivariate Cox regression analysis was performed. The risk score was calculated as follows: Risk score = ∑βi × expRNAi. Receiver operating characteristic (ROC) curve analysis was used to evaluate prognostic signature.

### Survival analysis of hub MDGs Analysis of Prognostic Value

The survival R package was used to conduct survival analyses based on hub MDGs expression and overall survival (OS) or disease-free survival (DFS) of patients based on TCGA-BC dataset.

### Gene set enrichment analysis (GSEA)

According to continuous BEND5 mRNA expression, biological functions and pathways were analyzed by GSEA software (v4.1.0) based on TCGA-BC dataset including 19712 genes. Gene sets with |Normalized Enrichment Score (NES)| > 1, Nominal *P* value < 0.05, and False Discovery Rate (*q*) < 0.05 were considered statistically significant.

### Cell culture and transfection

MDA-MB-231, ZR75-1 and HEK293T cells were incubated at 37 °C and 5% CO_2_ incubator using DMEM complete culture medium (containing 10% FBS and 1% penicillin-streptomycin solution). According to the manufacturer's instructions, vectors were transfected into cells. The reagents for transfection of plasmids were VigoFect reagent.

### Cell proliferation, migration and invasion assays

Cell proliferation were measured by CCK8 reagent following the manufacturer's instructions. For colony formation assays, cells were inoculated in 6-well plate with density of 2000 cells per well. Cell culture was terminated until visible colony formation (about 2 weeks). Cells were fixed with 4% paraformaldehyde solution and stained with 0.1% crystal violet solution. The number of colonies larger than 1.5 mm in diameter was calculate. Cell migration ability was detected by Wound-healing. After the cell density reached 80-90%, a pipette head was used to make a scratch. Floating cells were washed with PBS. After 16 h culture, photos were taken again at the initial location to calculate the distance of cell migration. Cell invasion were detected by Transwell assays. Briefly, 200 μL cell suspension with serum-free was added into an upper chamber with matrixgel. After cultured in an incubator at 37 ℃ for 16 h, the cells were fixed by 4% paraformaldehyde and stained with 0.1% crystal violet solution for 30 minutes. The number of invaded cells was calculated using Image J software.

### Co-immunoprecipitation (Co-IP)

Transfected Cells were lysed with 500 μL lysis buffer and then immunoprecipitated overnight at 4 °C with anti-FLAG or MYC-agarose beads. The agarose beads were collected and washed three times with lysis buffer and eluted in SDS sample buffer. For endogenous protein interaction assays, the protein extract was immunoprecipitated by antibody or control serum (Santa Cruz Biotechnology). The precipitated protein was isolated and eluted by SDS-PAGE according to the manufacturer's instructions.

### Analysis of tumor growth and metastasis in mice

All animal experiments were approved by the Institutional Animal Care Committee at Beijing Institute of Biotechnology. For subcutaneous model assay, 5 × 10^6^ MDA-MB-231 cells stably carrying shBEND5 vector or control vector were subcutaneously inoculated into right flank of six-week-old female nude mice. One month later, the nude mice were euthanized at the specified time. The resected tumor was stored in liquid nitrogen, and the length and width of the visible tumor were recorded using calipers. The tumor volume was calculated according to the following formula: volume = (longest diameter × shortest diameter^2^)/2. For orthotopic model of fat pad injection assay, 5 × 10^6^ MDA-MB-231 cells stably expressing BEND5 shRNA or control shRNA were injected into the inguinal fat pad of six-week-old female nude mice. One month later, the mice were imaged using the IVIS200 Imaging System (Xenogen Corporation, USA) and tumor cells in mice body was quantified with bioluminescence assay.

For tumor metastasis assay, 1 × 10^7^ MDA-MB-231 cells stably carrying shBEND5 vector were injected into the tail vein of each nude mice. One month later, the mice were imaged using the IVIS200 Imaging System. All lung metastases were examined histologically after euthanasia.

### Statistical analysis

The software GraphPad Prime 8.0 and Image J were used for statistical analysis and image processing of the experimental data. Student's *t* test was used to compare data between two groups, and analysis of variance (ANOVA) was used to compare several groups. Student's *t* test and Mann-Whitney test were used to analyze the relationship between clinicopathological characteristics and BEND5 in BC patients. All experiments were independently repeated for 3 times. Data were expressed as mean ± SD. *P* < 0.05 was defined as statistically significant difference.

## Results

### Identification of six hub MDGs in BC

**Figure 1:**
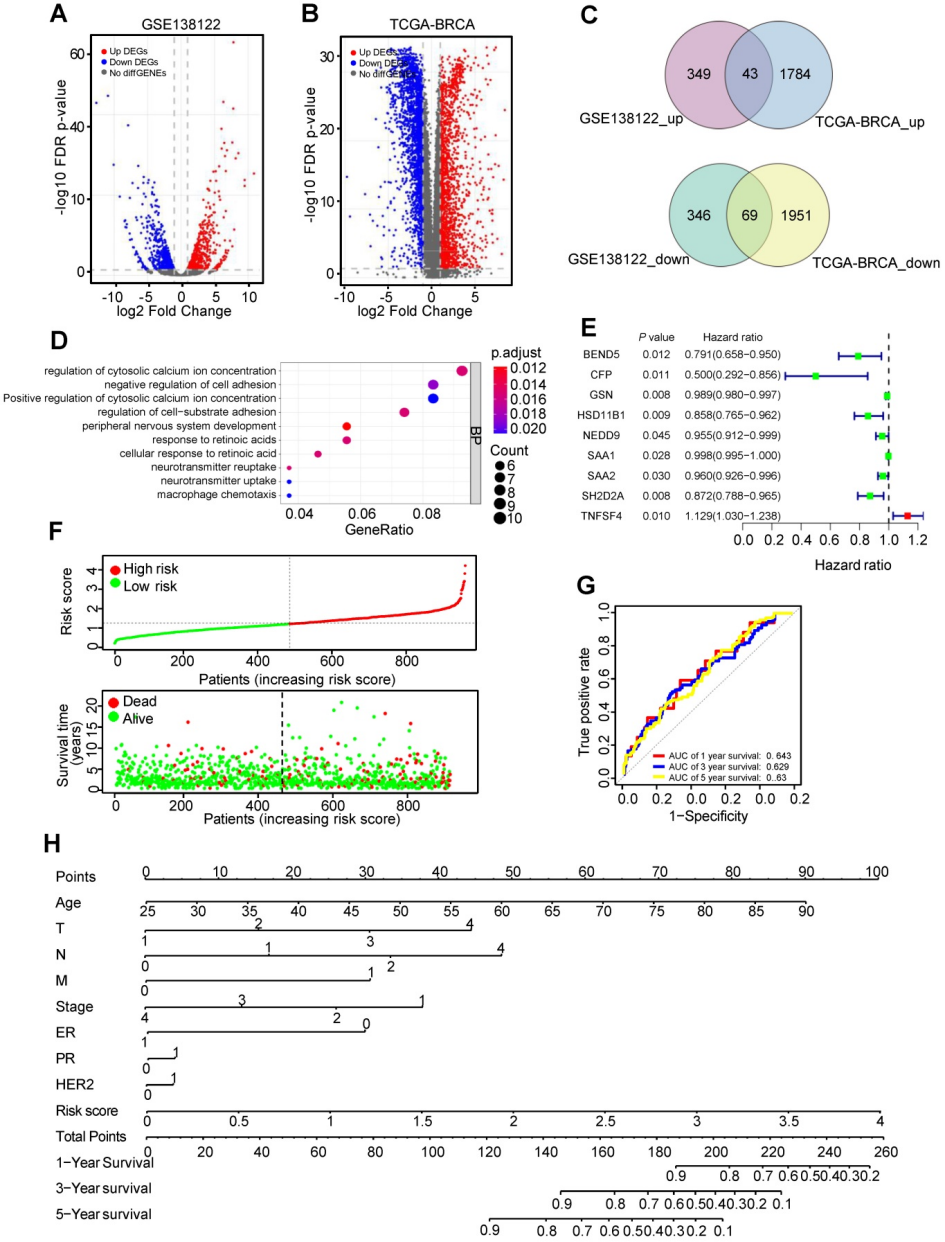
** Identification of metastasis driver genes (MDGs) and prognostic signature in BC. (A)** The volcano plot showing differentially expressed genes (DEGs) in MDA-MB-231 and its lung metastatic subpopulations from GSE138122 dataset. **(B)** The volcano plot showing DEGs in 112 paired BC tissues and adjacent tissues from TCGA-BC dataset. **(C)** The common differentially up/down-expressed genes identified as MDGs. **(D)** Top 10 enriched GO-BP pathways based on the MDGs. **(E)** The forest plot exhibiting MDGs that significantly correlates with overall survival (OS) based on univariable Cox regression analysis. **(F)** The distribution of risk score and survival status of BC patients from TCGA-BC dataset. **(G)** ROC curve plotted for the prognostic model with 1-, 3- and 5-year OS in BC patients. **(H)** The comprehensive nomogram for 1-, 3- and 5-year overall survival prediction of BC patients.

The human metastatic breast cancer cell line MDA-MB-231 and its lung metastatic subpopulations from GSE138122 dataset were analyzed. In this dataset, 392 upregulated and 415 downregulated DEGs were identified in MDA-MB-231 metastatic subpopulations compared with parental groups (Figure 1A and Supplementary Table 1). In TCGA-BC dataset, compared with adjacent normal tissues, 1827 DEGs were upregulated and 2020 DEGs were downregulated in 112 paired BC tissues (Figure 1B and Supplementary Table 2). There were 44 common upregulated DEGs and 69 common downregulated DEGs were identified as MDGs (Figure 1C). To explore the function of these MDGs, Biological Process (BP) of Gene Ontology (GO) terms was performed (Supplementary Table 3). GO-BP analysis showed that negative regulation of cell adhesion and regulation of cell-substrate adhesion were enriched (Figure 1D). These results indicated that some MDGs might play an important role in breast cancer metastasis.

Univariable Cox regression analysis was used to identify nine MDGs and BC patients' outcomes in TCGA-BC dataset (Figure 1E). Subsequently, multivariate Cox regression analysis constructed six-mRNA based prognostic signature, including BEND5, hydroxysteroid 11-beta dehydrogenase 1 (HSD11B1), neural precursor cell expressed, developmentally down-regulated 9 (NEDD9), serum amyloid A2 (SAA2), SH2 domain containing 2A (SH2D2A), and TNF superfamily member 4 (TNFSF4). Prognostic risk score formula was as follows: (-0.156 × BEND5 - 0.09 × HSD11B1 - 0.04 × NEDD9 - 0.01 × SAA2 - 0.09 × SH2D2A + 0.11 × TNFSF4). Among these six hub MDGs, BEND5 variable had the highest weight in the formula, suggesting that BEND5 may play an important part in metastasis and prognosis of BC patients.

In addition, according to the median risk score, the 959 BC patients were divided into low risk and high risk group. Besides, the distribution of risk score and corresponding OS data were plotted (Figure 1F). As shown in the figure, patients with higher risk score have shorter OS time and higher death rate. The area under the curve (AUC) value for prognostic characteristics of 1-year, 3-year, and 5-year survival were 0.643, 0.629, and 0.63, respectively, indicating a certain predictive effect (Figure 1G). Moreover, we integrated prognostic features, including age, T (tumor size), N (node status), M (metastasis), stage, ER (estrogen receptor), PR (progesterone receptor), HER2 (human epidermal growth factor receptor-2), and risk score, and developed the comprehensive nomogram to predict 1-, 3-, and 5-year OS of BC patient (Figure 1H).

### The validation of BEND5 and other five MDGs in TCGA-BC dataset

**Figure 2:**
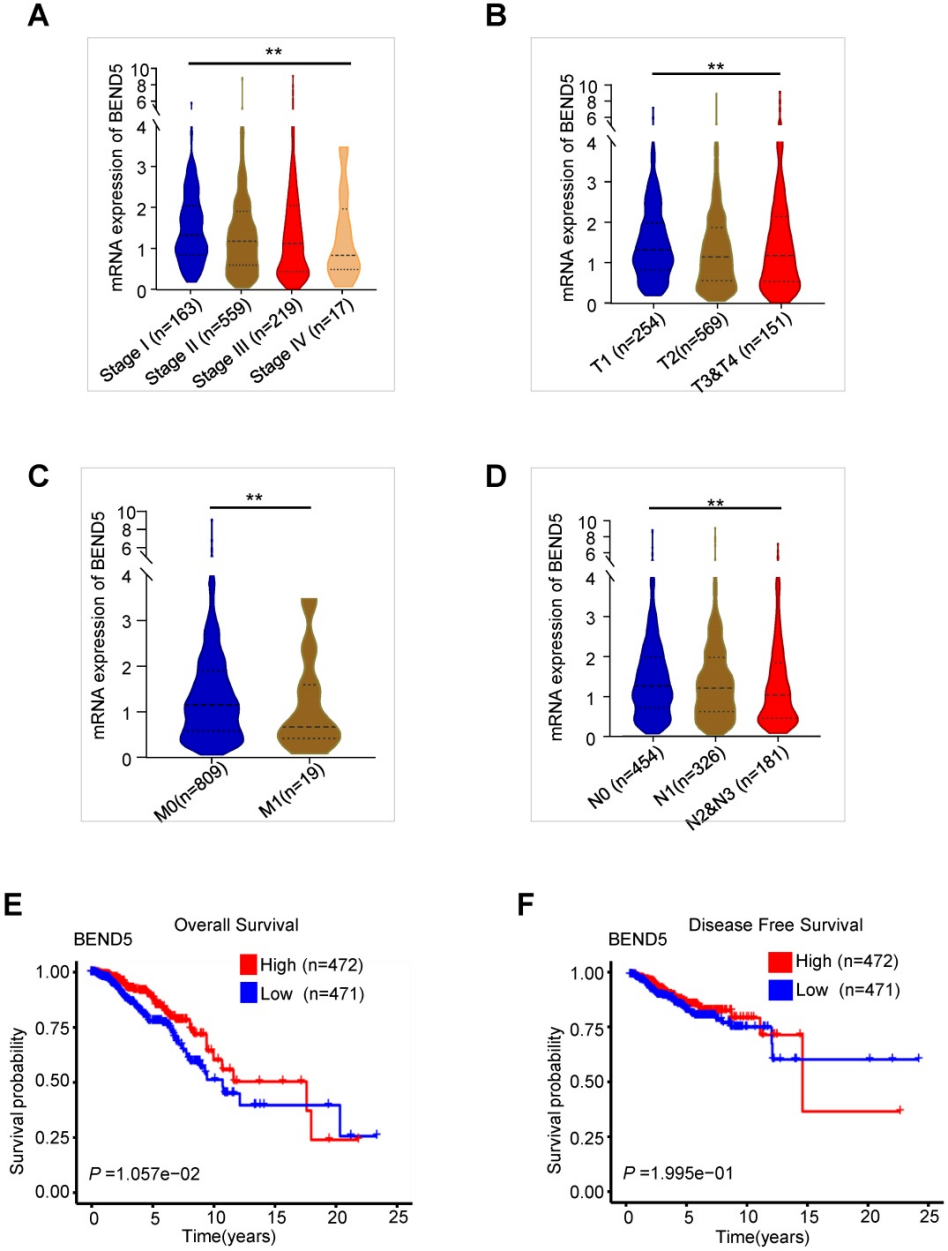
** The validation of BEND5 in TCGA-BC dataset. (A-D)** The associations between BEND5 mRNA expression and clinicopathological features in BC patients from TCGA-BC dataset. **(E and F)** The association between BEND5 gene expression and overall survival (E) and disease-free survival (F) in BC patients from TCGA-BC dataset.

We analyzed the association between these MDGs and the pathological features of BC patients in TCGA-BC dataset. Compared to those with early clinical stage BC, BEND5 mRNA level was significantly reduced in patients with advanced stage (Figure 2A). BEND5 mRNA expression was also decreased in patients with larger tumor size, lymph node metastasis as well as distant metastasis (Figure 2B-D). The associations between other five hub MDGs and clinical stage were also analyzed. HSD11B1 and NEDD9 mRNA levels were decreased in those with advanced clinical stage (Figure S1A-B). There were limited clinical values of SAA2, SH2D2A, and TNFSF4 mRNA level because of non-consistent expression level or non-significance in different clinical stages (Figure S1C-E).

In the first 15 years of BC, high BEND5 mRNA expression was significantly associated with longer OS in BC patients (Figure 2E). After 15 years, due to very few samples, the analysis may be inaccurate. In addition, a trend of association between high BEND5 mRNA expression and longer DFS was found although the result was not statistically significant (Figure 2F). The associations between other five hub MDGs and prognosis of BC patients were also analyzed. HSD11B1 and NEDD9 mRNA levels were positively correlated with longer OS, and TNFSF4 mRNA level was negatively correlated with longer OS in BC patients (Figure S2A-C). There was no significant correlation between other two hub MDGs and OS in BC patients (Figure S2D-E).

We further explored the relationship between these six MDGs and BC patients' outcomes in infiltrating ductal carcinoma or infiltrating lobular carcinoma. However, except for HSD11B1 and SH2D2A in infiltrating ductal carcinoma, other MDGs did not have statistically significant clinical outcome (Figure S2F-G), possibly due to small sample size.

### BEND5 suppresses BC cell proliferation, migration and invasion *in vitro*

**Figure 3:**
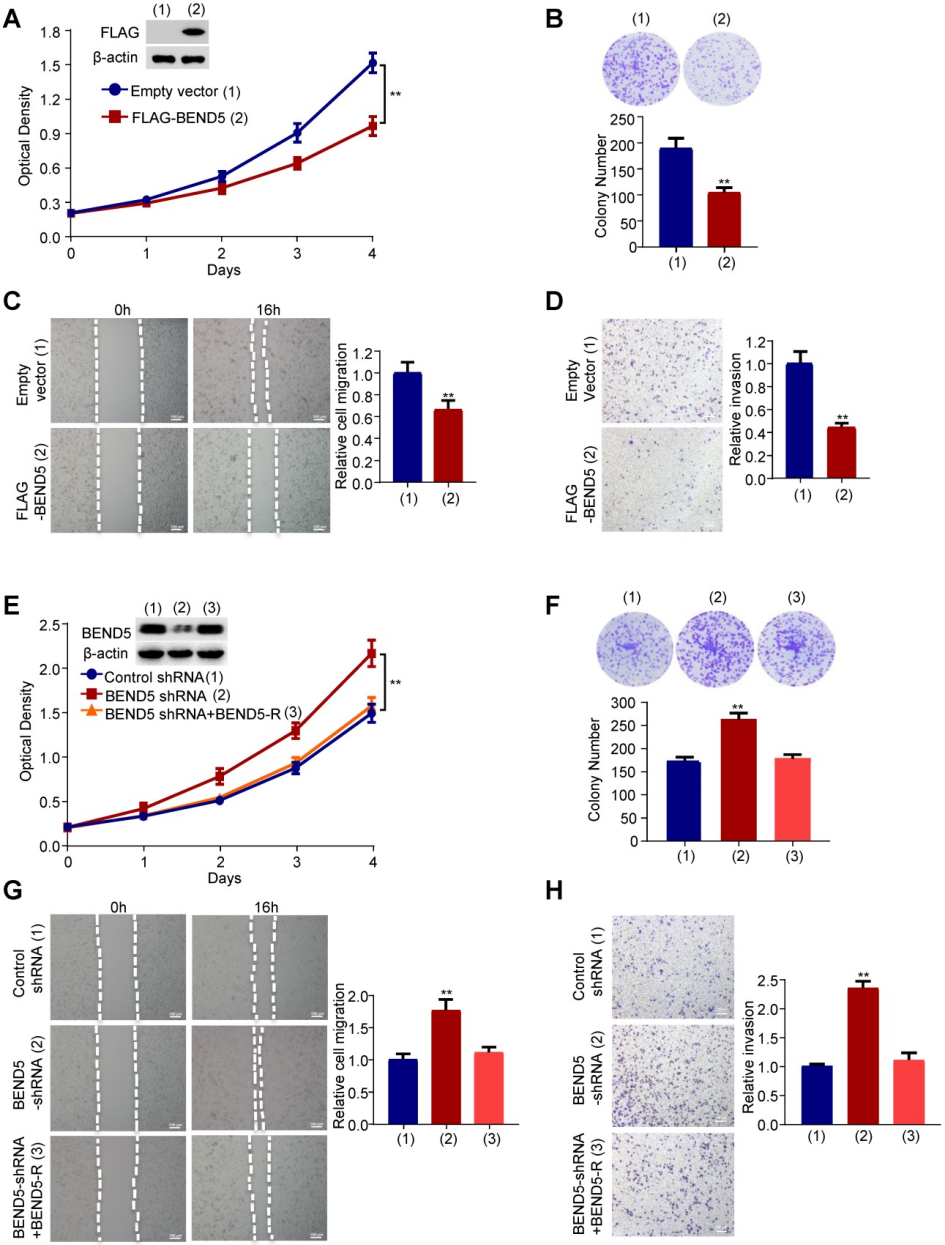
** BEND5 suppresses proliferation, migration and invasion in MDA-MB-231 cells. (A)** MDA-MB-231 cells were transfected with FLAG-tagged BEND5 or empty vector and cultured for a specified time. CCK8 assays were used to detect cell numbers, and immunoblot was used to detect the expression of BEND5 in MDA-MB-231 cells. β-actin was used as a loading control. **(B)** Colony formation assays for MDA-MB-231 cells transfected as in (A). **(C and D)** Wound-healing assays and transwell assays for MDA-MB-231 cells transfected as in (A). **(E)** MDA-MB-231 cells were transfected with control shRNA, BEND5 shRNA or BEND5 shRNA plus shRNA-resistant BEND5 (BEND5-R). CCK8 assay was used to detect cell numbers, and immunoblot was used to detect the expression of BEND5 in MDA-MB-231 cells. **(F)** Colony formation assays for MDA-MB-231 cells transfected as in (E). **(G and H)** Wound-healing assays and transwell assays for MDA-MB-231 cells transfected as in (E). Data shown are mean ± SD of triplicate measurements with similar results (*P < 0.05, **P < 0.01 versus empty vector).

Next, we examined the effect of BEND5 on proliferation, migration and invasion of breast cancer cells. Cell proliferation and colony formation assays revealed that FLAG-tagged BEND5-overexpressing MDA-MB-231 cells grew slower compared with empty vector-containing cells (Figure 3A & B). Wound-healing and Transwell assays showed that BEND5 overexpression could inhibit the migration and invasion of MDA-MB-231 cells (Figure 3C & D). Conversely, BEND5 silencing promoted the proliferation, migration and invasion of MDA-MB-231 cells (Figure 3E-H). Reexpression of BEND5 in the BEND5 knockdown cells rescued these effects. Similar results were obtained in ZR75-1 breast cancer cells (Figure S3). These data suggested that BEND5 has ability to inhibit BC cell proliferation, migration and invasion *in vitro*.

### BEND5 blocks Notch signaling-induced BC cell proliferation, migration and invasion as well as EMT-related gene expression

**Figure 4:**
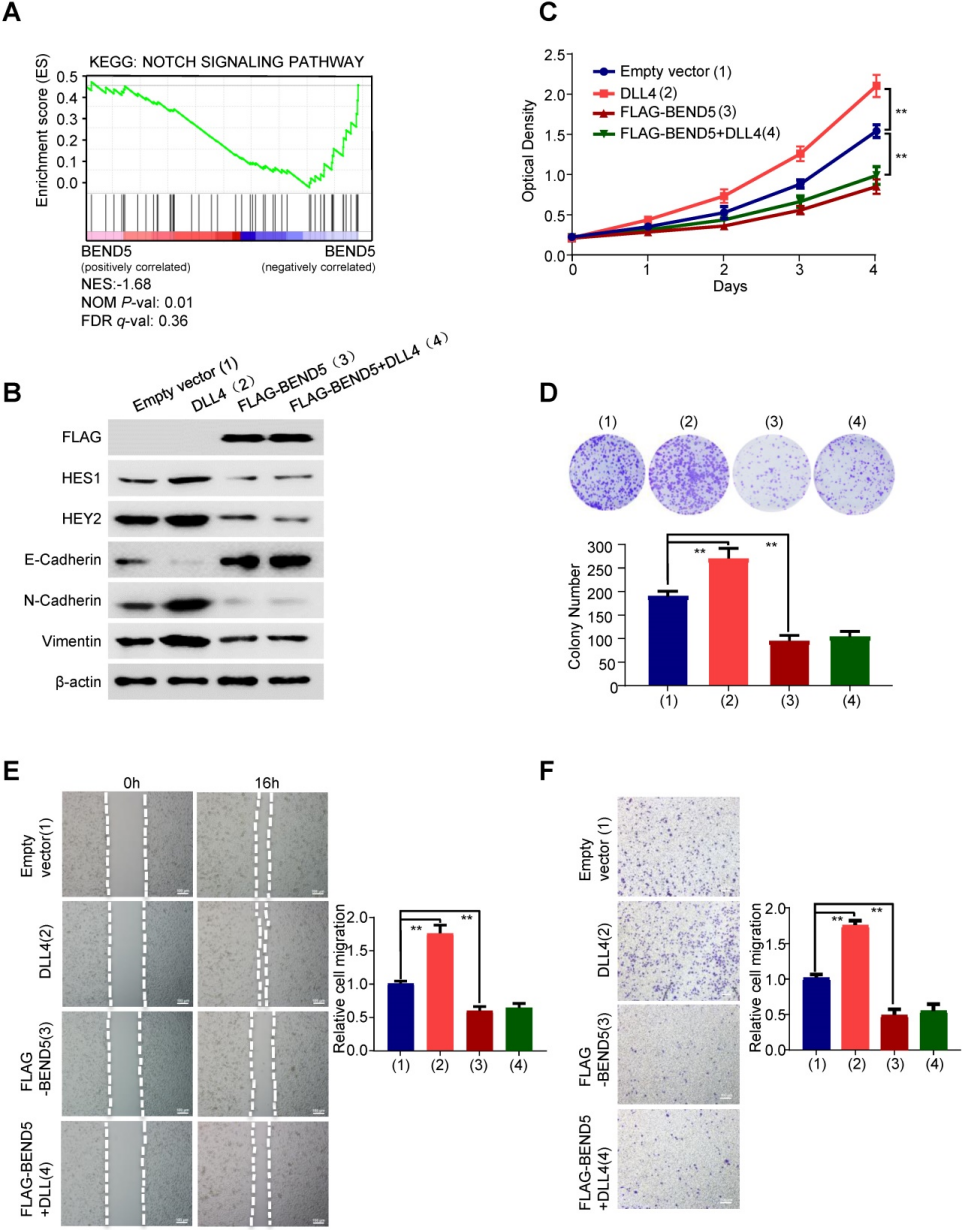
**BEND5 eliminates Notch signaling-induced BC cell proliferation, migration and invasion. (A)** GSEA was conducted to predict the function of BEND5 in BC. **(B)** MDA-MB-231 cells were transfected with empty vector or FLAG-tagged BEND5 and treated with/without Notch signaling activator DLL4 (10 ng/ml). Immunoblot was used to detect Notch pathway downstream targets and EMT-related proteins. **(C and D)** CCK8 assays and colony formation assays for MDA-MB-231 cells transfected and treated as in (B). **(E and F)** Wound-healing assays and transwell assays for MDA-MB-231 cells transfected and treated as in (B). Data shown are mean ± SD of triplicate measurements with similar results (*P < 0.05, **P < 0.01).

To explore the mechanism underlying the function of BEND5 on breast cancer. Gene Set Enrichment Analysis (GSEA) was performed (Figure 4A). The result revealed that BEND5 expression levels were negatively correlated with Notch signaling-related genes. Thus, we treated FLAG-tagged BEND5-overexpressing or empty vector-containing MDA-MB-231 cells with Delta-like ligand 4 (DLL4), which activates Notch signaling. As reported in other studies, compared with control group, DLL4 increased expression of Notch signaling downstream target genes HES1 and HEY2 14, 15 (Figure 4B). Epithelial-to-mesenchymal transition (EMT) is one of major mechanisms for BC metastasis and can be activated by Notch signaling 16. As previously reported, DLL4 decreased the epithelial marker E-cadherin expression, and increased the mesenchymal marker N-cadherin and Vimentin expression in MDA-MB-231 cells (Figure 4B). Importantly, BEND5 overexpression abrogated the effect of DLL4 on expression of Notch signaling downstream target genes and EMT-related genes. Similar results were observed in ZR75-1 cells (Figure S4A). BEND5 overexpression could also abrogate DLL4-promoted proliferation, migration and invasion of MDA-MB-231 cells (Figure 4C-F). Similar results were also observed in ZR75-1 cells (Figure S4B-E). These data suggested that BEND5 suppresses BC cell proliferation, migration and invasion through interrupting Notch signaling.

### BEND5 inhibits Notch signaling via binding to RBPJ

**Figure 5:**
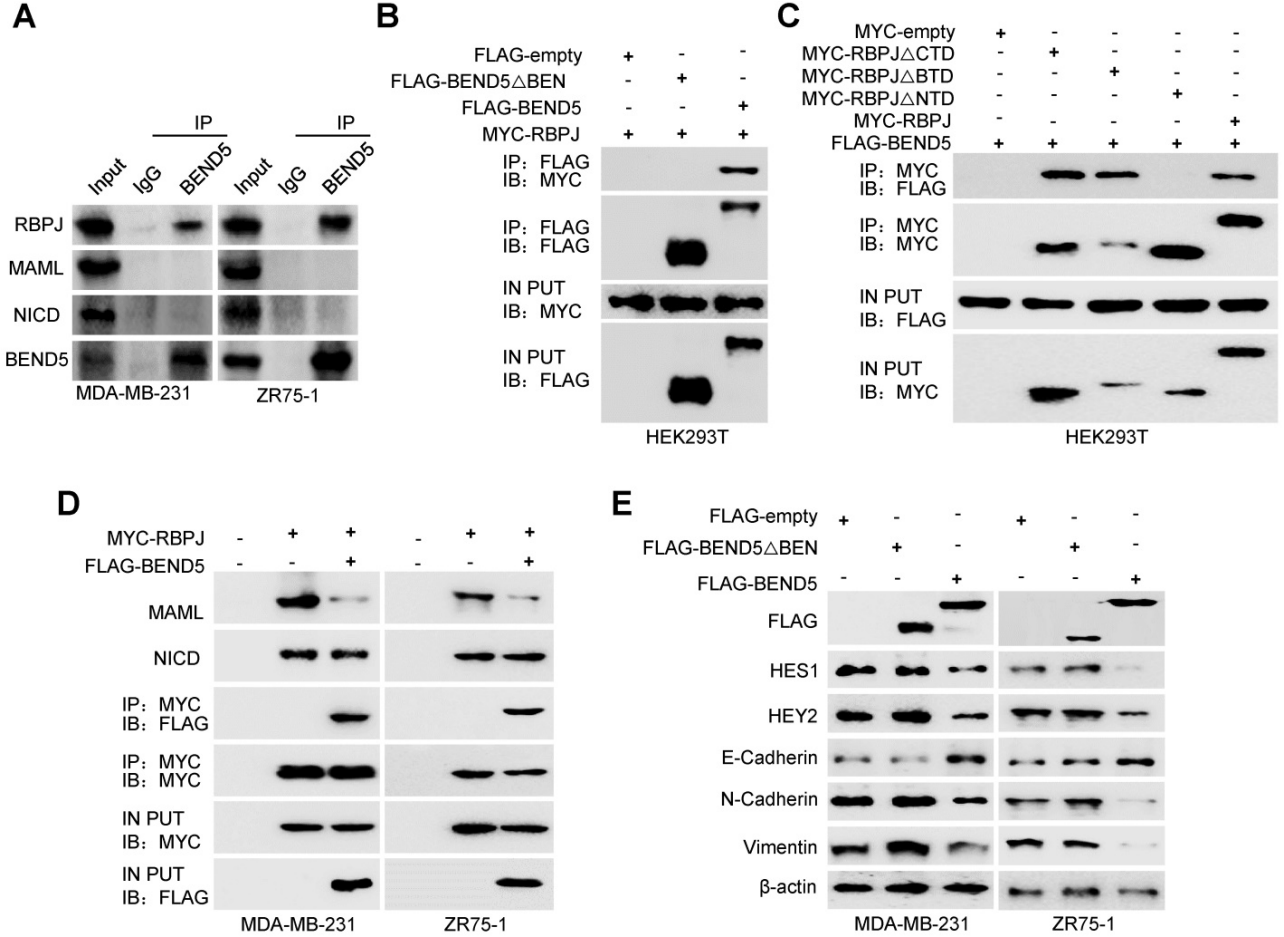
** BEND5 inhibits Notch signaling via binding to RBPJ. (A)** MDA-MB-231 or ZR75-1 cells were immunoprecipitated with anti-BEND5 or normal IgG, and the precipitates were analyzed by immunoblot with the indicated antibodies. **(B)** HEK293T cells were co-transfected with MYC-RBPJ and FLAG-BEND5 or FLAG-BEND5 △BEN as indicated. Cell lysates were immunoprecipitated with anti-FLAG, followed by immunoblot with anti-MYC. **(C)** HEK293T cells were co-transfected with FLAG-BEND5 and MYC-RBPJ, MYC-RBPJ △CTD, MYC-RBPJ △BTD, or MYC-RBPJ △NTD. Cell lysates were immunoprecipitated with anti-MYC, followed by immunoblot with anti-FLAG. **(D)** Co-IP analysis of MDA-MB-231 cells and ZR75-1 cells transfected with FLAG-tagged BEND5 and MYC-tagged RBPJ. Cell lysates were immunoprecipitated with anti-MYC, followed by immunoblot with the indicated antibodies. **(E)** Immunoblot was used to detect Notch pathway downstream targets and EMT-related proteins in MDA-MB-231 and ZR75-1 cells transfected with empty vector, FLAG-BEND5, or FLAG-BEND5 △BEN. Data shown are mean ± SD of triplicate measurements with similar results (*P < 0.05, **P < 0.01).

Since BEN domain could interact with RBPJ in *Drosophila* according to previous study 17, we speculated that RBPJ may be a potential BEND5 interaction partner in human cancer cells. In MDA-MB-231 and ZR75-1 cells, the Co-IP of endogenous proteins showed that BEND5 could interact with RBPJ, but not NICD and MAML (Figure 5A). Co-IP assays further showed that removal of BEN domain of BEND5 eliminated the interaction with RBPJ, and NTD domain of RBPJ was also required for this interaction (Figure 5B & C). Next, we tested how BEND5 regulates Notch signaling through RBPJ. The role of RPBJ as an transcriptional activator was mediated by the NICD-RBPJ-MAML ternary complex during Notch signaling activation 18. Intriguingly, BEND5 overexpression could interrupt the RBPJ/MAML interaction, but not the RBPJ/NICD interaction (Figure 5D). Moreover, the BEND5 mutant, which lacks BEN domain and fails to interact with RBPJ, also failed to regulate the expression of HES1, HEY2, and EMT-related proteins (Figure 5E). These results indicated that BEND5 has ability to interrupt the RBPJ/MAML interaction and inhibit Notch signaling via binding to RBPJ.

### Knockdown of BEND5 promotes BC tumor growth and metastasis in nude mice

**Figure 6:**
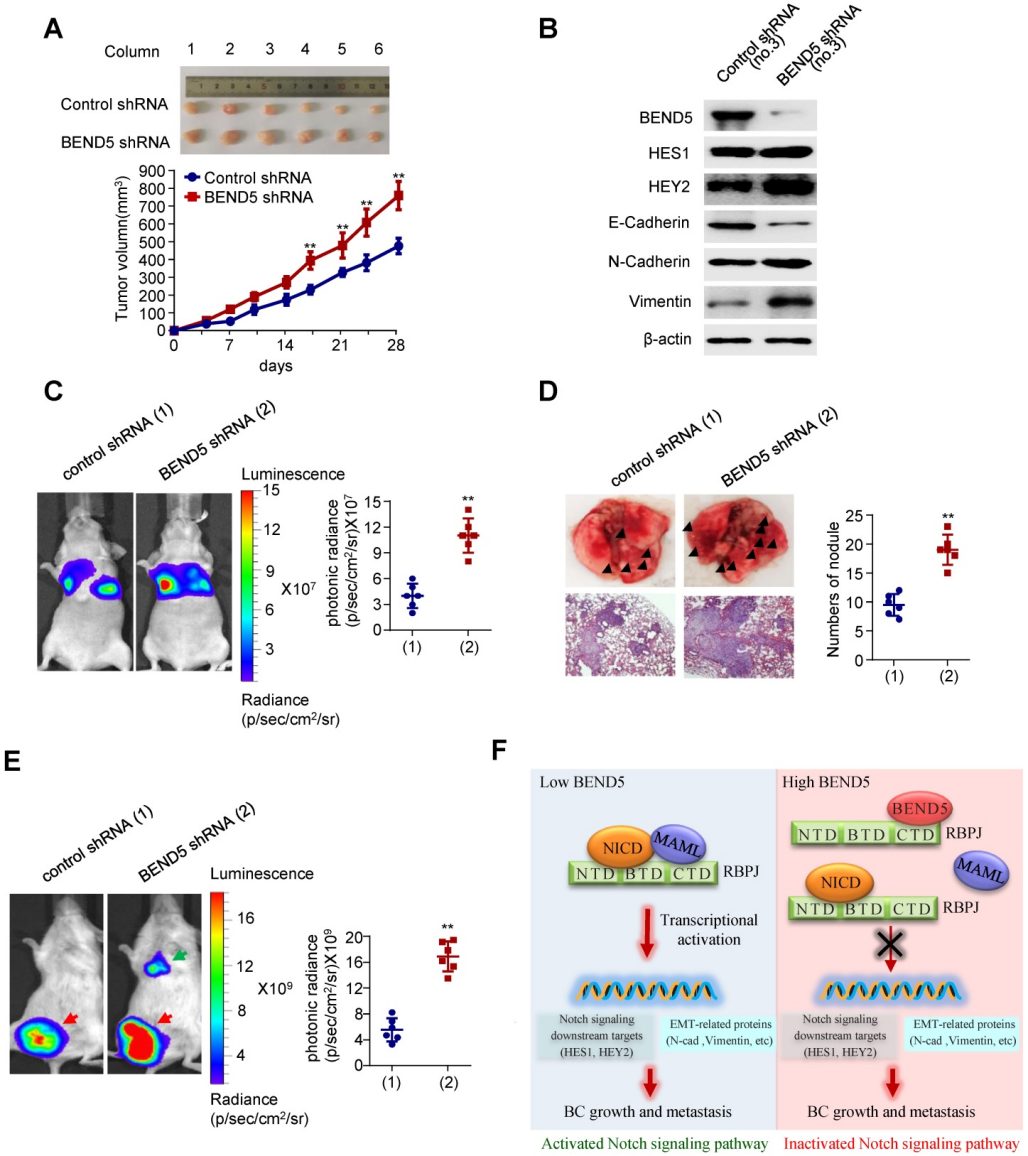
** Knockdown of BEND5 promotes BC tumor growth and metastasis in mice. (A)** MDA-MB-231 cells stably transfected with BEND5 shRNA or control shRNA were injected subcutaneously in the right flank of nude mice, and tumor volume was measured with vernier-caliper at the indicated times. **(B)** Immunoblot analysis of representative excised tumor tissues from (A). **(C)** Representative bioluminescence image at 30 days of nude mice injected by tail vein with MDA-MB-231 cells expressing firefly luciferase and the indicated constructs (n = 6). The luminescence signal is represented by an overlaid false-color image with the signal intensity indicated by the scale (right panel). **(D)** Representative lung tissues and H&E-stained sections of the lung tissues from (C). The number of tumor nodules are shown (right panel). Data are shown as mean ± SD (n = 6) (*P < 0.05, **P < 0.01 versus control shRNA). **(E)** MDA-MB-231 cells stably transfected with BEND5 shRNA or control shRNA were injected into mammary fat pad on the right side of nude mice (n = 6). Representative bioluminescence images are shown one month after injection. Red arrow indicates tumor growth and green arrow tumor metastasis. Tumor growth was compared between MDA-MB-231 cells expressing BEND5 shRNA and control shRNA (**P < 0.01 versus control shRNA). **(F)** A proposed model for BEND5 modulation of BC growth and metastasis. BEND5 inhibits Notch signaling by disrupting MAML-RBPJ interaction via competing with MAML for binding RBPJ.

To examine the effect of BEND5 on BC tumor growth *in vivo*, we subcutaneously injected MDA-MB-231 cells stably expressing BEND5 shRNA or control shRNA in the right flank of each nude mice. Compared with control shRNA, BEND5 knockdown in MDA-MB-231 cells significantly promoted tumor growth (Figure 6A). As expected, HES1, HEY2, N-cadherin, and Vimentin expression was increased, and E-cadherin expression was decreased in MDA-MB-231 tumor with BEND5 knockdown (Figure 6B).

In addition, we explored whether BEND5 regulates BC metastasis* in vivo*. MDA-MB-231 cells stably expressing BEND5 shRNA or control shRNA were injected into nude mice through the tail vein. The results showed that the luminescence signals in the lung region of mice in BEND5 shRNA group were significantly stronger than those in control group (Figure 6C). The number of tumor nodules in the lung region of mice in BEND5 shRNA group was greater than that in control group (Figure 6D). As mentioned above, there is no statistical significance although there is a trend of association between high BEND5 mRNA expression and longer DFS. The discrepancy between the clinical data and the animal data may be that DFS includes recurrence-free and metastasis-free survival or large clinical samples are needed to determine the exact association between BEND mRNA expression and DFS.

Finally, to better simulate tumor growth *in vivo*, we also injected MDA-MB-231 cells stably expressing BEND5 shRNA or control shRNA in the fat pad of each mouse. The tumor growth was detected with fluorescence intensity. The results showed that compared with control shRNA, tumors grew more rapidly in MDA-MB-231 tumors stably expressing BEND5 shRNA (Figure 6E). Taken together, these data suggested that BEND5 knockdown promotes BC tumor growth and metastasis *in vivo*.

## Discussion

In this study, six hub BC metastasis driver genes, including BEND5, HSD11B1, NEDD9, SAA2, SH2D2A, and TNFSF4, were identified by bioinformatics analysis. Among these genes, BEND5 plays the most important role in BC metastasis. Low BEND5 expression level is correlated with advanced stage, larger tumor size, lymph node, and distant metastasis. Moreover, BEND5 suppresses BC cell proliferation, migration, invasion and metastasis *in vitro* and *in vivo*. We further showed that BEND5 can interrupt Notch signaling through binding to RBPJ. BEND5 reduces expression of the Notch signaling downstream genes HES1 and HEY2, which were responsible for BC growth and metastasis 19, 20. BEND5 also elevates expression of the EMT-related protein E-cadherin, and reduces that of the EMT-related proteins N-cadherin and Vimentin. Overall, our study shows for the first time that BEND5 is a tumor suppressor gene related to BC growth and metastasis.

Activation of Notch signaling pathway was identified as one of main factors of BC cell survival, proliferation and metastasis 21-23. Various ligands reported by a number of studies, including DLL1 and DLL4, can stimulate Notch signaling pathway and have become important therapeutic targets for BC 24, 25. Disrupting the interaction between DLL4 and Notch1 induces tumor cell apoptosis and inhibits cell proliferation and EMT in breast cancer 26. Notch signaling has carcinogenic activity mainly through the formation of NICD-RBPJ-MAML ternary complex 27, 28. Our study found that BEN domain of BEND5 interacts with NTD domain of RBPJ, thereby preventing formation of RBPJ/MAML transcriptional activation complex. According to previous studies, MAML binds to NTD domain of RBPJ 29, 30, suggesting that BEND5 and MAML competitively bind to RBPJ. Consistent with these results, the BEND5 mutant which lacks BEN domain fails to regulate the expression of Notch signaling downstream proteins and EMT-related proteins.

In addition, we identified that four hub genes (HSD11B1, NEDD9, SAA2, and SH2D2A) were negatively correlated with BC metastasis and one hub gene TNFSF4 was positively correlated with BC metastasis by bioinformatic prediction. However, previous studies considered five hub genes as poor-prognostic factors in cancer patients, suggesting that bioinformatics analysis is a predictive tool for confirmation by molecular experiments. For example, HSD11B1, one of the other five hub genes in this study, maintains glucocorticoid concentration and predicts poor outcomes in patients with clear cell renal cell carcinoma 31. Single nucleotide polymorphisms (SNPs) in HSD11B1 may be associated with breast cancer among postmenopausal women 32. NEDD9 expression was shown to be positively correlated with metastasis and poor prognosis in triple-negative BC patients 33. Serum amyloid A 2 (SAA2) was known as a tumor-related marker and promoted Lewis lung carcinoma cell metastasis 34, 35. SH2D2A, also known as T cell-specific adaptor (TSAd), was required for vascular endothelial growth factor recepter 2 (VEGFR2)/c-Rous sarcoma (c-Src) interaction and c-Src activation, and promoted vascular permeability in tumor vessels 36, 37. Tumor necrosis factor ligand superfamily 4 (TNFSF4) is a cytokine that inhibits apoptosis and promotes chemoresistance in lung adenocarcinoma 38. The detailed biological function of these genes in breast cancer remains to be investigated.

## Supplementary Material

Supplementary figures.

Supplementary tables.
