# Supplementary material for: Interaction between BEND5 and RBPJ suppresses breast cancer growth and metastasis via inhibiting Notch signaling
Source: Int J Biol Sci. 2022 Jun 27;18(10):4233–44. doi: 10.7150/ijbs.70866 (PMC9274485; doi:10.7150/ijbs.70866)
Supplement: Supplementary file 1 — Supplementary figures. [file ijbsv18p4233s1.pdf]

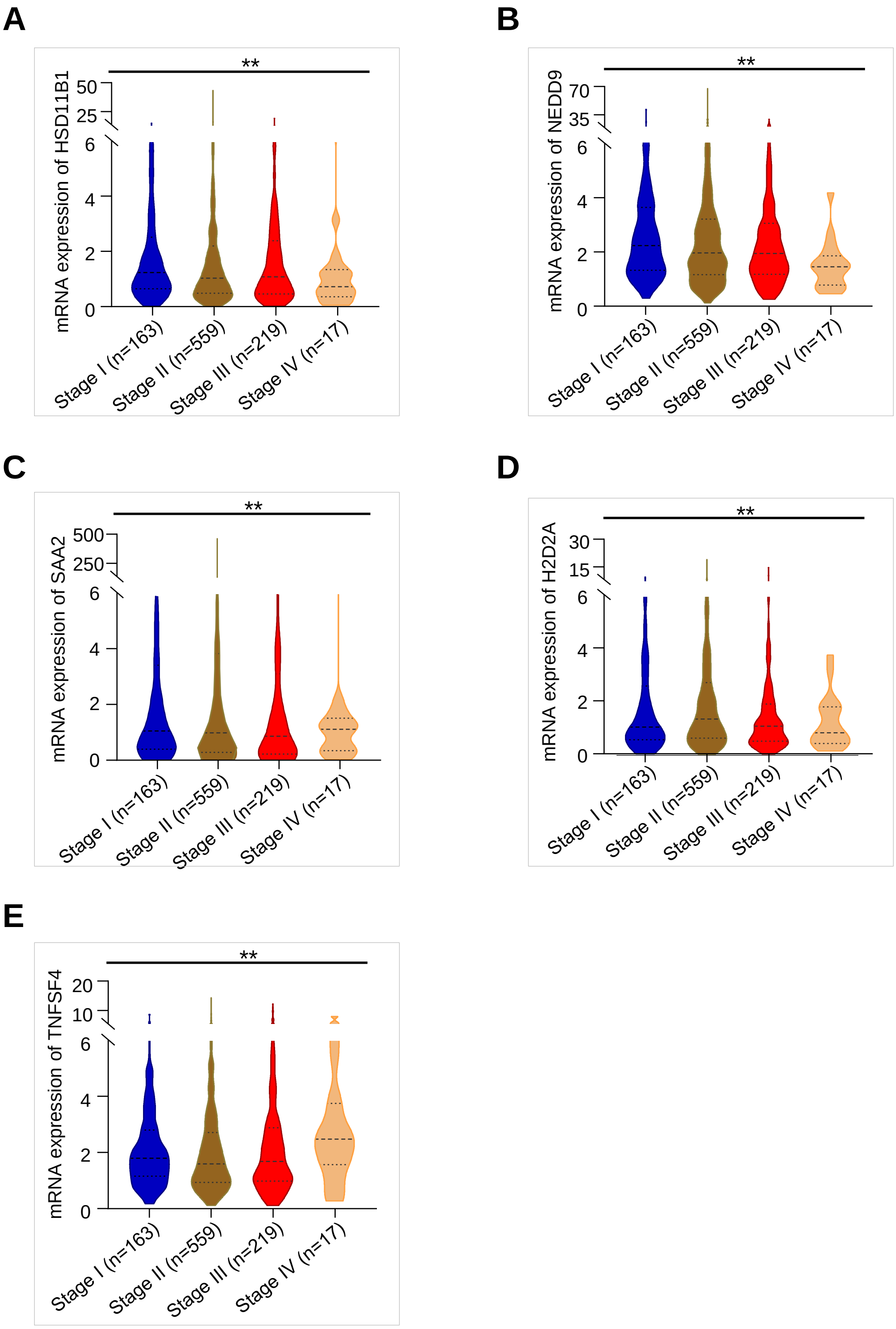

Figure S1. The validation of five hub genes in BC patients from TCGA-BC dataset. (A-E) The association between the mRNA expression level of HSD11B1 (A), NEDD9 (B), SAA2 (C), H2D2A (D) or TNFSF4 (E) and clinical stage in TCGA-BRCA dataset.

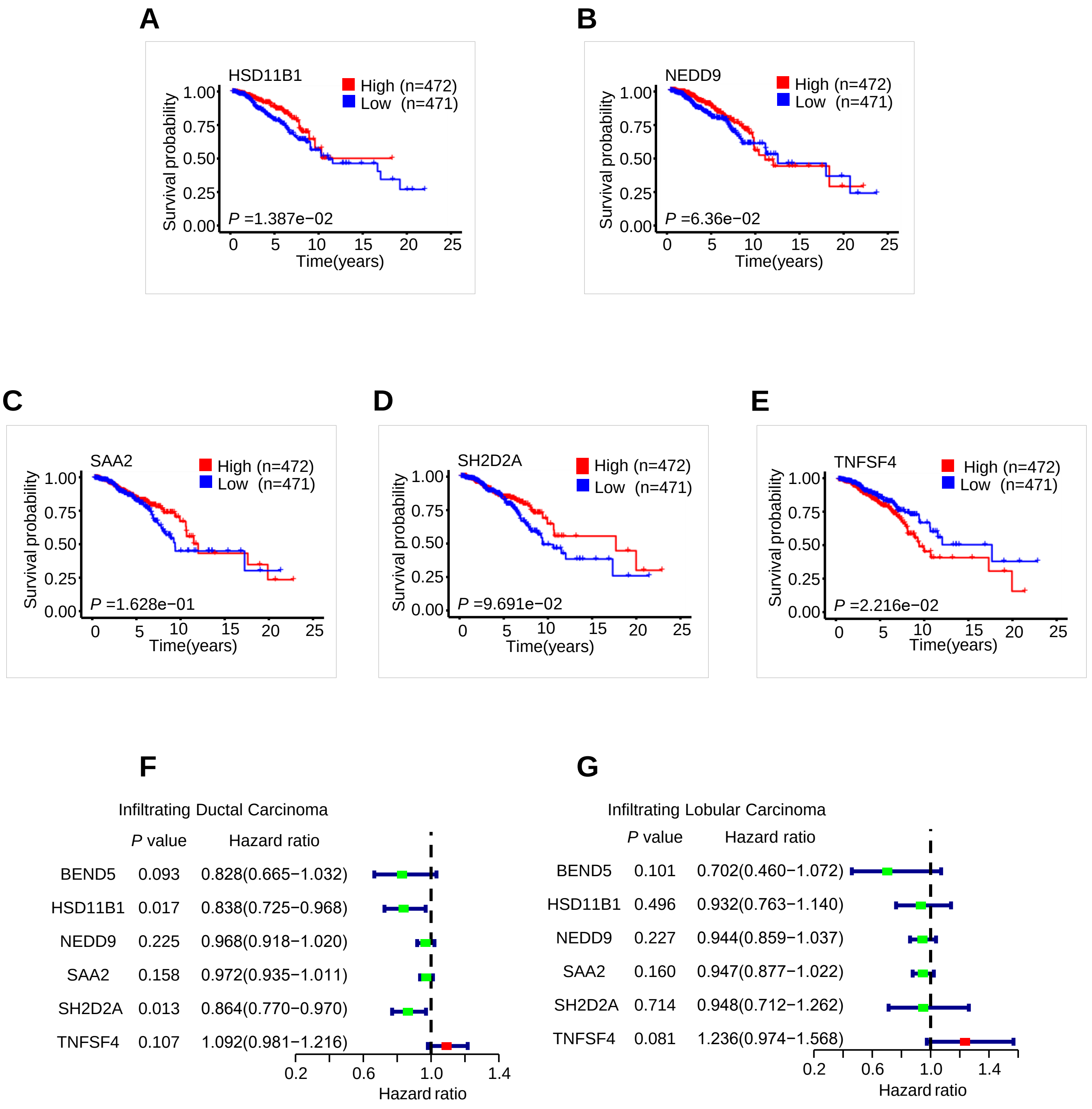

Figure S2. The association between five hub genes and clinical outcome in BC patients. (A-E) The associations between HSD11B1 (A), NEDD9 (B), SAA2 (C), H2D2A (D) or TNFSF4 (E) and overall survival in BC patients from TCGA-BC dataset. (F-G) The forest plot exhibiting the correlation between six MDGs and overall survival in infiltrating ductal carcinoma or infiltrating lobular carcinoma patients from TCGA-BC dataset based on univariable Cox regression analysis.

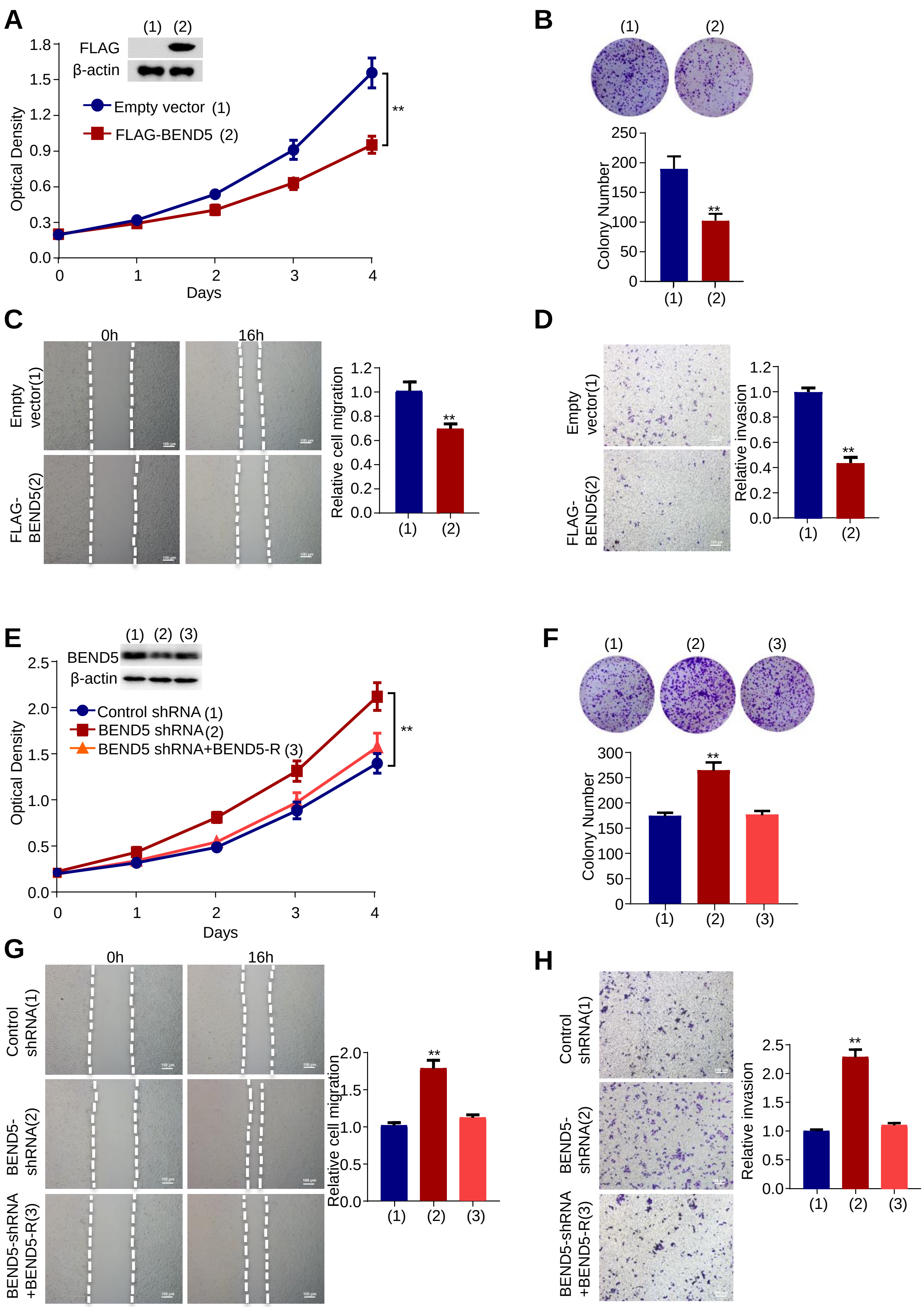

A
(1) (2)
FLAG
β-actin
Empty vector (1)
FLAG-BEND5 (2)
Optical Density
Days
**
B
(1) (2)
Colony Number
**
C
0h
16h
Empty vector(1)
FLAG-BEND5(2)
Relative cell migration
**
D
Empty vector(1)
FLAG-BEND5(2)
Relative invasion
**
E
(1) (2) (3)
BEND5
β-actin
Control shRNA (1)
BEND5 shRNA (2)
BEND5 shRNA+BEND5-R (3)
Optical Density
Days
**
F
(1) (2) (3)
Colony Number
**
G
0h
16h
Control shRNA(1)
BEND5-shRNA(2)
BEND5-shRNA +BEND5-R(3)
Relative cell migration
**
H
Control shRNA(1)
BEND5-shRNA(2)
BEND5-shRNA +BEND5-R(3)
Relative invasion
**

Fig S3. BEND5 suppresses proliferation, migration and invasion in ZR75-1 cells. (A) ZR75-1 cells were transfected with FLAG-tagged BEND5 or empty vector and cultured for a specified time. CCK8 assays were used to detect cell numbers, and immunoblot was used to detect the expression of BEND5 in MDA-MB-231 cells. β-actin was used as a loading control. (B) Colony formation assays for ZR75-1 cells transfected as in (A). (C and D) Wound-healing assays and transwell assays for ZR75-1 cells transfected as in (A). (E) ZR75-1 cells were transfected with control shRNA, BEND5 shRNA or BEND5 shRNA plus shRNA-resistant BEND5 (BEND5-R). CCK8 assay was used to detect cell numbers, and immunoblot was used to detect the expression of BEND5. (F) Colony formation assays for ZR75-1 cells transfected as in (E). (G and H) Wound-healing assays and transwell assays for ZR75-1 cells transfected as in (E). Data shown are mean $\pm$ SD of triplicate measurements with similar results (**P* < 0.05, ***P* < 0.01 versus empty vector).

Figure S4

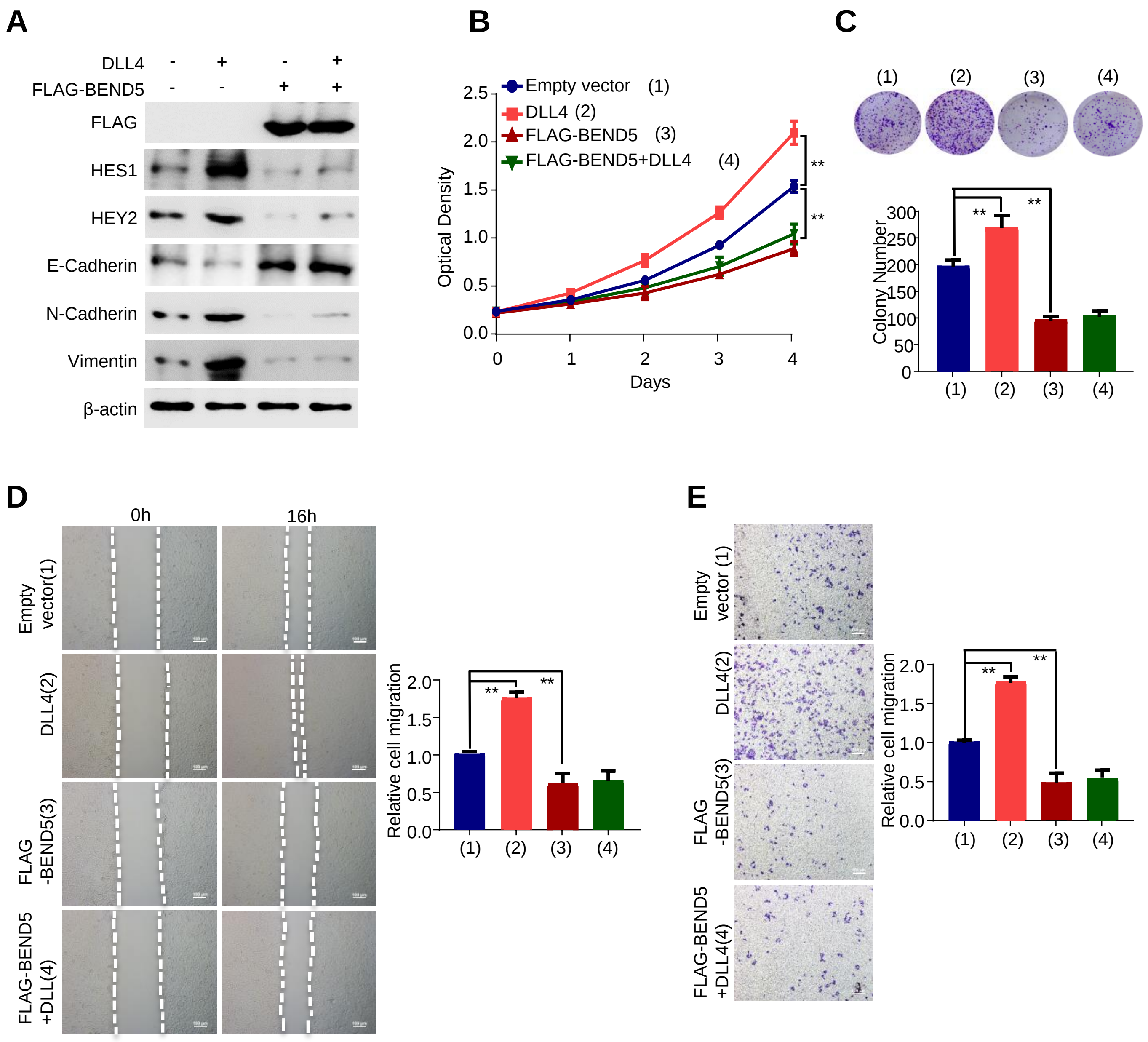

Fig S4. BEND5 inhibits Notch signaling-induced BC cell proliferation, migration and invasion. (A) ZR75-1 cells were transfected with empty vector or FLAG-tagged BEND5 and treated with/without Notch signaling activator DLL4 (10 ng/ml). Immunoblot was used to detect Notch pathway downstream targets and EMT-related proteins. (B and C) CCK8 assays and colony formation assays for ZR75-1 cells transfected and treated as in (A). (D and E) Wound-healing assays and transwell assays for ZR75-1 cells transfected and treated as in (A). Data shown are mean ± SD of triplicate measurements with similar results (**P* < 0.05, ***P* < 0.01).
